# A Medical Article Publishing Club for Junior Doctors: A Quantitative and Qualitative Analysis

**DOI:** 10.7759/cureus.3701

**Published:** 2018-12-07

**Authors:** Samson O Oyibo, Seema O Brij

**Affiliations:** 1 Internal Medicine, Peterborough City Hospital, Peterborough, GBR

**Keywords:** medical article publishing, thematic analysis, satisfaction survey, junior doctors' education

## Abstract

Introduction

There is an increasing expectation for junior doctors to have a publication. However, there is not much help provided to bridge the gap between project completion or case reporting and getting published. Our previous study demonstrated that junior doctors felt that involvement in medical article publishing contributes to learning and that it is an effective teaching method. Junior doctors also agreed that it is difficult to get published. Based on this study we started a medical article publishing (MAP) club. The aim of this study was to assess user satisfaction with the MAP club.

Materials and methods

Questionnaires were sent to 12 doctors who obtained advice from the MAP club since its inception in December 2017. The questionnaire contained two questions: (1) how useful they found the advice and guidance received and (2) how likely they were to recommend the MAP club to their colleagues. The 10-point Likert scale responses were analyzed quantitatively. Written comments and suggestions were analyzed qualitatively by thematic analysis.

Results

Respondents gave scores of either 9 or 10 to both questions indicating that they found the advice and guidance received very useful and that they were very likely to recommend the MAP club to their colleagues (weighted scores 9.75 and 9.92, respectively). The thematic analysis revealed three main themes: (1) positive features of the MAP club, (2) what participants gained from the MAP club and (3) recommendations for the future. The majority of respondents commented that the club was useful, helpful, supportive, and informative. More than a third commented that the club provided encouragement, confidence, practical tips, learning experience, and the opportunity to publish. A similar proportion asked that we keep the club going and provide dedicated teaching sessions.

Conclusions

Junior doctors feel that the MAP club has positive features and has contributed to their learning, education, and publishing skills. A proportion of them would also like to receive formal teaching sessions concerning medical article publishing. This study has highlighted the benefits and importance of establishing a medical article publishing club for junior doctors.

## Introduction

Having peer-reviewed articles published in medical journals is very important for career progression for junior doctors in many medical specialties [1, 2]. Not only does getting published provide junior doctors the opportunity to share educational experiences, provoke intellectual debate, and change practice. It also makes an important contribution to the scoring system for Physician Specialty Training Level 3 (ST3) recruitment. This covers recruitment to Specialty Training programmes in England, Scotland, and Wales, and also Northern Ireland for clinical genetics, medical oncology, and palliative medicine [3].

Getting a poster or presentations at regional or national meetings as well as full publications (be it research, audit report, or case report) has been seen as a sign of commitment to a chosen specialty. In fact, being first author of two or more PubMed-cited publications will provide a candidate with full marks for the publication section in the ST3 application [4].

It has been noted in the undergraduate medical student community that the number of publications produced is far less than the number of curricular faculty-mentored and student-run scholarly research projects being carried out by medical students. Lack of mentorship and student self-motivation may be the main reasons behind this production-outcome mismatch [5]. Could this be the same for junior doctors who also carry out a significant number of scholarly projects during their busy work schedules?

Despite the growing importance of medical article publishing to both junior doctor education and junior doctor career progression, there is no accompanying inclusion of a formal medical article publishing training module into the junior doctor training curriculum. There is lack of published data relating to article publishing by junior doctors and the use of a medical article publishing club to assist junior doctors with article publishing.

In a previous study we demonstrated that junior doctors feel that involvement in medical article publishing contributes to learning and education and that it is an effective teaching method [6]. However, a majority of junior doctors had not had adequate training or involvement in medical article publishing during their undergraduate medical training years. Worse still, it was the general view that it was difficult for them to get published as a junior doctor [6].

We therefore set up a medical article publishing (MAP) club to provide a forum for doctors to obtain advice and guidance concerning medical article publishing. Publications included scientific abstracts, posters, case reports, audit reports, and full articles. We aimed to assess user satisfaction with the MAP club as part of a quality of education improvement project.

## Materials and methods

The MAP club

Guidance consisted of providing advice on what, how, and where to publish, directions to additional resources, sample publications, case suggestions, and advice concerning journal-specific author instructions. Junior doctors could contact the instructors via email or physically to request for help with publishing. There were two dedicated instructors. The tips and resources provided by the club are demonstrated in the appendices (Appendix 1).

Study design

An anonymous, web-based survey was administered to medical doctors who had sought advice from the MAP club since its inception in December 2017. Questionnaire distribution and data collection were carried out prospectively over a six-month period. Ethics approval was sought through the Research & Development department of our institute. This survey did not require ethical approval on account of it being registered with our Quality, Governance and Compliance Department as a Quality of Education Improvement Project [6]. Participants were assured of strict anonymity and confidentiality during this survey.

Study participants

Twelve doctors sought advice concerning medical article publishing (case reports, abstracts, or full articles) over a six-month period. An email was sent out to each doctor asking whether they would participate in this survey and we received permission-replies from all 12 doctors.

Questionnaire and administration

The survey questionnaire was prepared online using SurveyMonkey [7]. We could not find any previously validated questionnaires so we created one to cater for this survey. The questionnaire contained two questions: (1) how useful they found the information, advice and guidance received from the club and (2) how likely they were to recommend the MAP club to their colleagues. Both questions required answers using the 10-point Likert scale (1 being not useful or very unlikely up to 10 being very useful or very likely, respectively). A comment and suggestion box was included. A web-link to the questionnaire was sent via email to the participants.

Data analysis

The responses to questions were analyzed quantitatively by expressing the percentages (and whole numbers) of responses to each question on the Likert scale. The weighted average score for both questions was calculated from “10”, which meant a majority of respondents found the information very useful and were very likely to recommend the club to a colleague, down to “1”, which meant a majority of respondents did not find the information useful and were very unlikely to recommend the club to a colleague, for questions 1 and 2, respectively [8].

The comments and suggestions were transcribed verbatim and analyzed qualitatively by the process of thematic analysis [9, 10]. The data was reviewed for initial codes, sub-themes and subsequently developed themes related to positive/negative features, what was gained, and any recommendations for the future progress of the MAP club. The raw data, sub-themes, and themes were continuously reflected upon to ensure credibility and trustworthiness of this survey [11].

## Results

Twelve doctors have obtained advice and guidance from the MAP club since its inception in December 2017. We received completed questionnaires from all participants (response rate of 100%).

Quantitative analysis

For question-1 concerning the usefulness of the information received from the MAP club, nine respondents (75%) gave a score of 10 and three respondents (25%) gave a score of 9, giving a weighted average of 9.75 for that question. For question-2 concerning how likely it was that they would recommend the club to a colleague, 11 respondents (92%) gave a score of 10 and one participant (8%) gave a score of 9, giving a weighted average of 9.92 for that question. Therefore, the majority of the doctors found the information they received very useful and were very likely to recommend the MAP club to their colleagues.

Qualitative analysis

Twelve respondents provided comments and suggestions, which were recorded verbatim. These comments and suggestions are shown in Table 1.

**Table 1:** A list of the 12 participants’ comments and suggestions

Participants’ comments/suggestions
“Was very helpful and in fact with more practical tips and information which has really helped me to start of my project and hopefully would be submitting for publication soon. Please continue to share your experience and knowledge which would be very valuable for people us who have limited experience in writing an article”
“The guide was very informative and has encouraged me to write medical papers.”
“As a junior doctor with very less experience in case reports, this was really helpful”
“As an International Medical Graduate, I believe it is a great platform to learn and gather experience about how to write/publish an article. I certainly believe MAP helped all of us to grow our own writing skills and gave the courage to submit our own writing in different international journals. I would suggest MAP to organize more meetings/seminar/teaching for medical professionals to help them improve their writing skills. Best of luck to MAP.”
“Very supportive”
“I'm satisfied and very impressed with the help I received from the Medical Article Publishing club towards the publication of my article. Keep up the good work.”
“Organise teaching sessions for junior doctors about how to publish a medical article.”
“Found it very useful for first time users.”
“It is very useful to have this club to get advice and help to write a paper. Also help me to learn and give confident to improve independent writing skills.”
“The opportunity it provides to young doctors like myself in terms of article publishing.”
“The club is excellent. There needs to be a visible presence within the hospital so that more people can use the services provided.”
“Very clear and helpful guidance as well as support throughout the whole process. Thank you so much, couldn’t have it done without you.”

Three major themes emerged from a thematic analysis: (1) positive features of the MAP club, (2) what was gained from the MAP club, and (3) the recommendations for the future. The themes and sub-themes are shown in Table 2. The process used for the thematic analysis is shown in the appendices (Appendix 2).

**Table 2:** Themes and sub-themes from the participants’ comments and suggestions

Positive features of the MAP club
Helpful
Useful
Supportive
Informative
Excellent service
What participants gained from the MAP club
Provided encouragement/courage to publish
Provided confidence to publish
Provided practical tips
Provided a learning experience
Provided the opportunity to publish
Recommendations for the future
Need for future teaching sessions
Keep the programme going
Need for visible presence

Theme 1 - Positive Features of the MAP Club

A majority of the respondents (83%) highlighted positive features of the MAP Club. Sub-themes related to this were demonstrated in the respondents’ comments and suggestions. Examples of these were: how excellent the service was, how helpful, how supportive, how useful, and how informative the service was.

Theme 2 - What Participants Gained from the MAP Club

A significant proportion of the respondents (42%) made positive comments and suggestions related to what they gained from using the service. Related sub-themes were demonstrated by comments such as: the service provided encouragement or gave them the courage and also provided them the confidence to be able to write their own medical article. Others mentioned that the club provided them with a learning experience, a much-needed practical tip, and also gave them that opportunity to contribute to a medical article publication.

Theme 3 - Recommendations for the Future

A significant proportion of the respondents (42%) made suggestions concerning improvements that can be made for the future. These were demonstrated by the following sub-themes: the need for the programme to keep going, the need for formal teaching sessions for junior doctors, and one participant mentioned the need for a physical presence, which we interpreted as the need for a physical forum or office where junior doctors can attend for advice and guidance when writing medical articles.

## Discussion

The results of this survey suggest that junior doctors feel the MAP club has several positive features with both learning and educational benefits. A proportion of them would also like to receive formal teaching sessions concerning medical article publishing.

Despite the growing importance of medical article publishing (abstracts, case reports, audit reports, etc.,) for junior doctors, there are no published reports relating to or evaluating the use of a medical article publishing club or forum to help junior doctors with medical article publishing during their busy clinical work. The junior doctor training curriculum does emphasize the need for junior doctors to be involved in audits, quality improvement projects, case presentations at national meetings, and research on one hand [12], and the ST3 recruitment panel emphasizes the importance of achieving peer-reviewed PubMed-cited publications on the other hand [3]. However, there is not much help provided to bridge the gap between project completion or case reporting and getting published.

There are published journal-specific instructions for authors (case reports, abstracts, research articles, etc.), and some journals have provided additional aid with medical article publishing [13, 14]. Despite this, junior doctors still find it difficult to get published during their training. This fact emphasizes the importance of mentorship and training, which could be provided by a curriculum-based medical article publishing club or forum.

Medical students have highlighted the benefits gained while writing case reports [15]. Another study reported that only a minority of students had written or presented case reports and that there were multiple educational values. The barriers to writing case reports and the importance of good mentorship and training were also highlighted in this study [16]. Participants in a writers’ workshop indicated they experienced lasting positive effects on observation, empathy, and future writing skills [17]. Our study quantitatively and qualitatively examined the value of a medical article publishing club to junior doctors working in a busy hospital.

Limitations of this study

Despite the favourable response rate, there are important limitations to this survey. Firstly, participants’ inherent interest in getting published could have biased their response to the questionnaire. A majority of the participants were able to write an article (abstracts, case reports or research articles), of which some have been published. It would be interesting to analyse feedback from a similar group of participants who were not able to write an article or get published despite receiving help from an MAP club. Secondly, the sample size was small, limited to one institution, and limited to the help received from just two instructors; therefore, the results of this survey may not be generalizable. Future studies should include a larger number of junior doctors who, after receiving help from the MAP club, are followed up to see if they do get published and go on get more than one publication. Future studies should also be multi-institutional, taking into account a larger number of physician-instructors and teaching methods over a longer time period. These important points can be incorporated into a collegiate/faculty-led national survey assessing junior doctors’ knowledge, attitude, support, and practice around medical article publishing, with the aim of providing a curriculum-based training module.

## Conclusions

Despite the limitations, this study is unique. It not only highlights the importance of medical article publishing to junior doctors but also, and more importantly, highlights the benefits and importance of establishing a medical article publishing club. Establishing such clubs or forums as part of the junior doctor training curriculum will aid in getting junior doctors published.

## Appendices

Appendix 1: The MAP club

**Table 3:** Tips and resources provided by the MAP club

Article type	Tips provided
Abstracts	Discuss a completed project
	Suggest relevant scientific meeting
	Guide with abstract writing instructions
	Constructive review of draft abstract and guide to submission
Posters	Discuss accepted abstract
	Offer poster templates and examples
	Constructive review of draft poster and guide to submission
Case reports	Finding an interesting/unusual case
	Discuss the case
	Suggest relevant journals
	Discuss journal author guide and specific instructions
	Provide sample/example case reports
	Help with literature review
	Constructive review of draft article
Original articles	Discuss a research report
	Suggest relevant journals
	Discuss journal author guide and specific instructions
	Provide sample/example research reports
	Help with literature review
	Constructive review of draft article
Other helpful resources	
Sun Z. Tips for writing a case report for the novice author. J Med Radiat Sci 2013;60(3):108-113. https://www.ncbi.nlm.nih.gov/pmc/articles/PMC4175810	
Kirthi V. How to write a clinical case report. RCP insight. Royal College of Physicians. 2011. http://www.bsaci.org/professionals/RCPhow_to_write_a_clinical_case_report.pdf	
Natarajan A. What, where, and how to publish. BMJ Careers [cited 05 May 2007]; Available from: http://careers.bmj.com/careers/advice/What,_where,_and_how_to_publish	
Faculty of library science. Suggestions for writing critical reviews of journal articles. University of Manitoba. May 2006. Available from: http://www.umanitoba.ca/libraries/units/dafoe/media/reviews_of_journal_articles.pdf	
Committee on publishing ethics (COPE). Code of conduct. Available from: https://publicationethics.org/resources/code-conduct	

Appendix 2: The thematic analysis process - raw data, codes, sub-themes, and final major themes

**Table 4:** Thematic analysis: generating codes and sub-themes Codes have been highlighted (bold and underlined text) in Stage 1

Stage 1 Examined comments/suggestions for codes		Stage 2 Generated codes	Stage 3 Generated sub-themes
1	“Was very helpful and in fact with more practical tips and information which has really helped me to start of my project and hopefully would be submitting for publication soon. Please continue to share your experience and knowledgewhich would be very valuable for people us who have limited experience in writing an article”	Very helpful	Helpful
		Practical tips	Provides practical tips
		Information	Informative
		Really helped	Helpful
		Continue to share experience and knowledge	Keep the program going
2	“The guide was very informative and has encouraged me to write medical papers”	Very informative	Informative
		Encouraged	Encourage/gave courage
3	“As a junior doctor with very less experience in case reports, this was really helpful”	Really helpful	Helpful
4	“As an International Medical Graduate, I believe it is a great platform to learn and gather experience about how to write/publish an article. I certainly believe MAP helped all of us to grow our own writing skills and gave the courage to submit our own writing in different international journals. I would suggest MAP to organize more meetings, seminar, teaching for medical professionals to help them improve their writing skills. Best of luck to MAP”	Platform to learn and gather experience	Learning experience
		Helped	Helpful
		Gave courage	Encourage/gave courage
		Organize more meetings/seminar/teaching	Need formal teaching sessions
5	“Very supportive”	Very supportive	Supportive
6	"I'm satisfied and very impressed with the help I received from the Medical Article Publishing club towards the publication of my article. Keep up the good work!”	Help I received	Helpful
		Keep up the good work	Keep the program going
7	“Organise teaching sessions for junior doctors about how to publish a medical article”	Organise teaching sessions	Need formal teaching sessions
8	“Found it very useful for first time users”	Very useful	Useful
9	“It is very useful to have this club to get advice and help to write a paper. Also help me to learn and give confidence to improve independent writing skills”	Very useful	Useful
		Club to get advice and help	Helpful
		Learn writing skills	Learning experience
		Gave confidence to improve writing skills	Provides confidence
10	“The opportunity it provides to young doctors like myself in terms of article publishing”	Opportunity to publish	Provides opportunity to publish
11	“The club is excellent. There needs to be a visible presence within the hospital so that more people can use the services provided”	Club is excellent	Excellent service
		Visible presence	Need for visible presence
12	“Very clear and helpful guidance as well as support throughout the whole process. Thank you so much, couldn’t have it done without you”	Helpful guidance	Helpful
		Support throughout process	Supportive

**Table 5:** Thematic analysis: re-examining and reflecting on the sub-themes

Sub-themes	Frequency	Comment numbers from Table 4 where sub-themes were found
Provides confidence	1	9
Provide practical tips	1	1
Encourage/give courage	2	2, 4
Helpful	6	1, 3, 4, 6, 9, 12
Useful	2	8, 9
Informative	2	1, 2
Keep the program going	2	1, 6
Need formal teaching sessions	2	4, 7
Supportive	1	5, 12
Learning experience	2	4, 9
Excellent service	1	11
Need for visible presence	1	11
Provides opportunity to publish	1	10
Total	25	

**Table 6:** Thematic analysis: developing the major themes from the sub-themes

Sub-themes	Developed major theme groups
Provides confidence	What they gained from the MAP club
Provide practical tips	What they gained from the MAP club
Encourage/give courage	What they gained from the MAP club
Helpful	Positive features
Useful	Positive features
Informative	Positive features
Keep the program going	Recommendations for the future
Need formal teaching sessions	Recommendations for the future
Supportive	Positive features
Learning experience	What they gained from the MAP club
Excellent service	Positive features
Need for visible presence	Recommendations for the future
Provides opportunity to publish	What they gained from the MAP club

**Table 7:** Thematic analysis: final major themes and frequency of use by participants

Major themes	Participant comment numbers from Table 4 containing these major themes	Number of participants providing these themes
Positive features of the MAP club	1, 2, 3, 4, 5, 6, 8, 9, 11, 12	10
What they gained from the MAP club	1, 2, 4, 9, 10	5
Recommendations for the future	1, 4, 6, 7, 11	5
